# Maternal Regret and the Myth of the Good Mother: A Psychosocial Thematic Analysis of Italian Women in a Patriarchal Culture

**DOI:** 10.3390/bs15111433

**Published:** 2025-10-22

**Authors:** Erika Iacona, Maria Masina, Ines Testoni

**Affiliations:** 1 Department of Philosophy, Sociology, Education and Applied Psychology, University of Padova, 35131 Padova, Italy; maria.masina@studenti.unipd.it (M.M.); ines.testoni@unipd.it (I.T.); 2 Department of Political Science, Law and International Studies, University of Padova, 35121 Padova, Italy

**Keywords:** motherhood regret, patriarchal culture, myth of the good mother, obstetrical violence, gaslighting, consciousness rising

## Abstract

Motherhood regret still constitutes a major taboo that limits the possibility of processing the negative exposure to being a mother. This qualitative study involved Italian women living both in Italy and abroad, where traditional patriarchal thinking remains influential. Sixteen women defining themselves as ‘regretful were interviewed to explore their experiences of regret, the changes following the birth of children, family and social support, and employment. The thematic analysis highlighted several recurring themes: the idealisation of motherhood and the hidden struggles it conceals; the guilt associated with feeling inadequate and the indifference of some fathers; the social pressure that compels women to conform to maternal expectations; the perception of being trapped in a predefined role; and the conflict between personal identity and the ideal of the “perfect mother.”. The findings reveal that maternal regret is deeply intertwined with internalised patriarchal norms, the myth of the “good mother,” and the social expectation of women’s self-sacrifice. Despite recognising these as cultural constructs, participants expressed feelings of guilt, anger, and inadequacy, intensified by the unequal division of domestic and parental responsibilities. This issue and the need for a revival of women’s consciousness-raising groups to open a space for dialogue on the topic in countries where patriarchy is still strong, such as Italy, are discussed.

## 1. Introduction

Regret is the sincere act of feeling repentance or remorse for one’s wrongdoings, often accompanied by a commitment to changing behaviour (Ruttenberg, 2022; Tudor et al., 2021). In cognitive social psychology, these kinds of sensations have been studied in the context of counterfactual thinking (Roese & Olson, 2014), which results in the mental process of imagining alternative scenarios to past events, considering how things might have turned out if different choices had been made. The “power of backward thinking” (Kahneman & Miller, 1986) leads people experiencing a currently unwanted or unfavourable condition to review their past actions by envisaging better outcomes they could have achieved, feelings of regret over disappointing past events, and missed opportunities (Fisher & Exline, 2010; Tangney & Dearing, 2003). A large body of literature shows that engaging in counterfactual thought often results in high levels of negative affect because it generates strong feelings of dissatisfaction, envy, and regret and leads individuals to view situations less favourably, reducing their self-efficacy and the will to initiate new ventures (Roese, 1997; Epstude & Roese, 2008).

The literature on the construct of regret is developed especially in restorative justice (Etzioni, 2000), where it is shown the complexity of the constellation of the implied emotions, as Thalberg (1963) described making a distinction between four aspects: “repentance” derived by choices in which there was no clear deliberation and without a plan to rectify the wrong action; “guilt” linked to the idea of having transgressed norms/laws with the risk of incurring punishment; “shame” related to actions even unintentional ones that cause social disdain; “regret” concerning events unrelated to one’s will and a consequent loss whose effects one rejects in their own life; “remorse” involving the will to change due to the non-acceptance of the effects of a choice. Among many other authors partly or wholly referring to this pattern, some of them point out the differences between remorse, guilt, and sorrow (Bassett et al., 2011); guilt and remorse (Taylor, 1996); remorse, apology, and mercy (Murphy, 2006); and “focused” remorse (feeling bad about a specific action) versus the global one, feeling like a bad person overall (Bassett et al., 2011). More specifically, as indicated by Landman (1993), we can say that regret is an emotion composed of both cognitive elements and affective aspects following what is perceived as loss, shortcoming, or mistake.

In the context of motherhood, the literature considers regret to be a complex sentiment that takes on all the above-mentioned elements, with the additional aspect of being considered taboo, especially from patriarchal perspectives (O’Reilly, 2019). The relationship between motherhood and regret has sometimes been addressed in religiously motivated literature, particularly in relation to abortion, where some studiesoften rooted in specific Christian traditionsframe intentional abortion as psychologically harmful (Lowe & Page, 2022). However, such works do not directly focus on motherhood regret itself. Instead, it is primarily feminist (Donath, 2015) and constructivist literature (Butler, 2002; Izhak & Aharoni, 2023; Yeo, 1999) that explore this issue, highlighting how the management of motherhood is shaped by cultural and social power dynamics rather than being exclusively linked to natural or biological factors (Dutta, 2024). It should be noted that the taboo surrounding regret has been reinforced by patriarchal ideologies and, in many cases, by religious discourses through the myth of the good mother (Abraham, 2019; Bartkowski & Read, 2003). Such discourses, however, vary across traditions and historical contexts; therefore, it is important to avoid treating them as uniform (Liu & Xu, 2024; Heffernan & Stone, 2021a; Mesman et al., 2016). In this context, as indicated by O’Reilly (2019), the image of motherhood has been assembled on the following axes: (1) “essentialisation”: all women want to be mothers, (2) “naturalisation”: all maternal abilities are inspired by motherlove and are innate, and (3) “idealisation”: all mothers find joy and purpose in motherhood. Specifically, the myth of the good mother (Douglas & Michaels, 2005) or “myth of mothering joy”assumes that the typical woman desires to be a mother in order to be happy, and this aspiration is the pivotal element of biological femininity, from which both mothering skills and fulfilment in personal scarifying for children naturally emerge (Izhak & Aharoni, 2023).

Traditional patriarchal culture has established that not being happy because of children and the sacrifice they require is unacceptable (Schafer, 2008). Such beliefs have been implemented by wage policies to support the family income that is mostly procured by men, relegating women to the role of housewife (Feasey, 2017; Foster & Clark, 2018; Hilbrecht et al., 2008; Williamson et al., 2022). From this binary ideology, they derive all the factors that maintain the gender gap between women’s and men’s pay and the following lower level of personal and professional opportunities of self-realisation (Horrell & Humphries, 1995), penalising women by undermining their levels of autonomy and freedom (Olalde-Mathieu et al., 2023). Because of this myth, feelings of inadequacy, guilt, and shame emerge in mothers who feel that they are not at all happy with their condition (Meeussen & Van Laar, 2018). Moore and Abetz (2018) show that the regret of becoming a mother is structured on several factors: (1) having had children with difficult temperaments or complex diagnoses, as this leads to greater difficulties in everyday life; (2) having a poor view of oneself as a parent due to a range of personality traits or disorders; (3) not feeling suited to motherhood and not coping with the losses and sacrifices it entails; and (4) not having planned the pregnancy or having experienced strong external pressure to pursue it.

In Italy, hegemonic masculinity is often reinforced by patriarchal traditions and has been described in the literature as being supported, at least in part, by certain strands of right-wing populist politics and aspects of Catholic education (Aloè et al., 2024; Giorgi, 2021; Pozzo, 2013; Tager & Good, 2005; Testoni, 2012). This situation is accompanied by significant forms of backlash aimed at slowing down or counteracting women’s emancipation (Melo Lopes, 2019). In the Italian context, many women still struggle to reconcile family, intimate relationships, and work, partly because of the wide gender gap in the labour market (Rubolino, 2023). Even when women are employed, research highlights persistent imbalances not only in wages but also in the disproportionate burden of unpaid and invisible care work, the so-called ‘double burden’ (Aziz, 2023; Patimo & Pereiro, 2017; Spagnoli et al., 2020; Väänänen et al., 2005).

Italy provides formal policies supporting parenthood, including maternity, paternity, and parental leave, as well as childcare allowances. Employed women are entitled to mandatory five-month maternity leave, while fathers have mandatory ten-day paternity leave, highlighting a clear disparity in caregiving responsibilities. Both parents can take optional parental leave, traditionally paid at 30% of their salary and usable within the child’s first twelve years; since 2024, additional months paid at 80% have been introduced, to be used within the first six years. Childcare bonuses and maternity allowances are also available for families, including adoptive and foster parents. However, despite these measures, limited paternal leave and persistent gender norms contribute to an unequal distribution of caregiving responsibilities, which can increase maternal stress, burnout, and feelings of regret.

Despite the variability of the Italian context, parents attribute great importance to the family unit. Mothers often remain the central figures in children’s growth and education, while fathers are traditionally associated with economic support. In recent years, however, increasing paternal involvement has been observed in educational practices and in everyday care (Bombi et al., 2011). Nevertheless, the contribution of civic and feminist organisations plays a significant role in reshaping the situation, as they strive to challenge the ambivalence of some fathers who, while expressing a desire to build relationships with their children marked by physical closeness and emotional intimacy comparable to those of mothers, simultaneously tend to reject these very dimensions (Magaraggia, 2012).

These social and patriarchal pressures also shape mothers’ psychological experiences, influencing phenomena such as maternal regret and parental burnout.

In recent years, research has increasingly focused on the psychological dimensions of maternal regret, particularly in relation to parental burnout (Piotrowski, 2021; Roskam et al., 2025). Historically considered rare and often associated with specific conditions or children with special needs, these phenomena are now recognised as more widespread, especially in highly developed and individualistic societies (Mikolajczak et al., 2017). This prevalence has been linked not only to social and cultural expectations but also to individual psychological processes, including perceived stress, perfectionism, and ruminative thinking. Parental burnout and regret affect both the well-being and quality of life of parents, influencing the parent–child relationship and potentially exacerbating feelings of inadequacy, guilt, and shame (Piotrowski et al., 2025; Roskam et al., 2025).

Traditionally, parenthood was supported by multigenerational networks and communal practices, which helped distribute the emotional and practical burdens of caregiving. Socioeconomic development, urbanisation, and the spread of nuclear families have increased parental isolation and responsibility, intensifying stress and the likelihood of experiencing regret (Piotrowski et al., 2025). From a psychological perspective, this suggests that maternal regret is not solely a product of social pressures or patriarchal norms but also emerges from the interaction between individual vulnerability, cognitive–emotional processes, and the challenges of contemporary parenting (Piotrowski et al., 2025; Roskam et al., 2025), and from the complex interplay between societal pressures and the mother’s internal emotional and cognitive world.

Maternal ambivalence, that is, the coexistence of positive and negative emotions, appears to be an inevitable phenomenon that emerges during the antenatal period, before the mother knows the child’s actual characteristics. Nonetheless, the idealisation of the early mother–child bond tends to exclude the narration of negative experiences, making it difficult to recognise and express these emotions (Raphael-Leff, 2010). From this perspective, it becomes clear that maternal regret is not solely a product of patriarchal norms but also reflects individual vulnerability, coping strategies, and the cognitive–emotional challenges of contemporary parenting.

## 2. Objectives

Although direct research on maternal regret in Italy is still lacking, some related studies have explored women’s motivations for remaining childfree (Lazzari & Charnley, 2016; Dal Ben, 2017) or the experiences of mothers reconsidering motherhood. The scarcity of research on maternal regret may be due to its lack of social acceptance in patriarchal cultures, where it challenges the idea that motherhood must always be desired, as the myth of the good mother dictates, resulting in an ‘invisible’ experience in social narratives (Sihto & Mustosmäki, 2021). Therefore, the main objective of this study was to delve into the experiences of Italian mothers who perceive regret over their motherhood, offering them a voice. Specifically, the investigation focused on the paths that led participants to become mothers, the daily difficulties of parenthood, changes resulting from motherhood, the relationship with the children, and the triggers of regret, strategies for managing this feeling, and the role of the social culture and of the intimate network.

We investigated the extent to which regret, often embedded in counterfactual thinking that limits a person’s developmental opportunities, can be influenced by increasing women’s awareness of the consequences of their choices regarding motherhood (Baron, 2000; Gleicher et al., 1995). From this perspective, we considered the usefulness of legitimising mothers to build a new vision of motherhood (Matley, 2020) and underlined the importance of allowing this experience to be talked about in a normalising context (Donath, 2015).

## 3. Materials and Methods

The study adopts a qualitative interpretative framework, using thematic analysis (Braun & Clarke, 2006) as its guiding methodological lens. This approach was chosen for its theoretical and methodological flexibility, which allows for both inductive coding and the integration of participants’ voices, making it particularly suitable for exploring the multidimensional (cognitive and emotional) experiences of participants in relation to maternal regret. It enables the identification, analysis, and interpretation of recurring themes without being constrained by a pre-existing theoretical model, while allowing both predefined and emerging themes to be explored as they arise during the analysis. Thematic analysis, therefore, provides a rigorous methodological structure while maintaining the flexibility necessary to capture the nuances of participants’ narratives (Pope, 2000).

Participants were informed of the objectives of the research and the methodology of analysis and gave their informed consent and permission to record, transcribe, and analyse the conversations. Despite the heterogeneity of participants in terms of country of residence, number of children, disability status, and socioeconomic background, recurring patterns and themes emerged across interviews. No new significant concepts appeared after the 14th interview, indicating that thematic saturation was achieved. Each participant was interviewed online for about 60 min via a semi-structured dialogue on the designated topics. The semi-structured interviews were based on a series of open-ended questions covering various topics: the path that led the participants to become mothers, the main difficulties they encountered in their daily lives and the strategies they employed to cope with them, the identification of predominant feelings related to motherhood and maternal regret, the meaning attributed to the role of mother, and the assessment of support at the marital, family, and social levels. A clear and well-defined structure was used as a starting point while allowing room for additional questions to further explore specific aspects. Given the sensitive nature of the topics addressed, the researchers informed participants that the interview would be interrupted in the event of high emotional distress. To process the texts, thematic analysis was used because of its flexible theoretical frame, utilising the Atlas.ti computer software, version 22 (ATLAS.ti Scientific Software Development GmbH, Berlin, Germany; Muhr, 1991), which better permits us to respect the phases outlined by Braun and Clarke (2006): familiarisation with the data, coding generation, reviewing themes, defining and naming themes, and finally, writing up the report. The codes were developed inductively from the data: two trained researchers independently reviewed the transcripts, generated initial codes, and then discussed discrepancies until a shared coding framework was agreed upon. This process ensured inter-coder reliability, further strengthened by the review of coding decisions and the emerging thematic structure by a third researcher. Thanks to this approach, recurring patterns and themes were identified in the interviews. Pseudonyms were utilised, and the quotations have been slightly altered to prevent any possibility of identification. The study complied with the ethical principles and code of conduct of the American Psychological Association and was approved by the Ethics Committee of the University of Padua (Protocol: 5376; Unique Number: 2647E8256547CD805B45A8519807531F).

### Participants

Since online forums and social media offer a space where mothers can share difficult experiences and break the silence on maternal regret (Fahlgren & Williams, 2023; Garncarek, 2020; Heffernan & Stone, 2021a; Moore & Abetz, 2018), we decided to recruit participants by a call on Facebook, in groups related to maternity, asking the members to take part in an interview. The recruitment post explicitly mentioned maternal regret, indicating that we were seeking women who had experienced feelings of regret regarding their role as mothers in order to ensure that participants self-identified with the phenomenon under investigation. As an exclusion criterion, we decided that mothers who had given birth less than 12 months prior were excluded due to the difficulties of this period of adaptation; thus, three new mothers could not participate. Sixteen Italian women between 28 and 49 years of age (X = 39.06; SD = 2.83) were involved. None of them had more than two children (X = 1.56: the youngest was 1 year old and the oldest 20; two children had disabilities); six (37.5%) lived abroad, while the remaining ten (62.5%) in Italy, among whom eight (80%) resided in the northern and two (20%) in the southern regions; ten (62.5%) were married, two (12.5%) cohabited with their partner, two were widows (12.5%), one (6.25%) was divorced, and one (6.25%) was separated. Regarding their educational qualifications, one (6.25%) had a middle school diploma, six (37.5%) had a secondary school diploma, eight (50%) had a university degree, and one (6.25%) had a doctorate (Table 1).

## 4. Findings

The analysis of the results revealed five thematic areas, which are summarised in Table 2.

### 4.1. First Thematic Area: The Idealised Mother: Pressures and Hidden Struggles

In this thematic area, the participants’ narratives illustrate the complexities of motherhood, including societal expectations, pressures from family and partners, and personal experiences of regret.

In the opinion of most participants, regret stems from the fact that motherhood is always portrayed in an idealised way by women and by society, omitting the more difficult aspects, as clearly expressed by Sara, a 35-year-old Italian married woman living in Zurich who, after the birth of her four-year-old son, realised how narratives on this issue are unreliable: “You become [a] mother because of dishonest messages. Mothers are not sincere, even with other women. I was not at all prepared for the life that was to come. Mothers closest to me my mother, my grandmother, my mother-in-law did not have any kind of honesty in bringing me their testimony. ‘It was all great and all natural!’ Then, when I investigated, I found out that for them, too, the story was completely different, with a suffering body, crying, haemorrhoids, hospitalisations”.

Others repented their decision because they were influenced by the pressures received from partners and birth families, as Paola (41 years old, living in northern Italy with her husband and two children, ages 4 and 2) narrated, affirming that she wanted to become a mother because of the type of education she had received: “I really regretted becoming a mother when I realised that this decision was the result of the idea of motherhood inculcated by society through the family: ‘you are a mother, and this is the most important thing you can do in your life’. This concept needs to be reformulated, changed, and eliminated. I go to the toy shop with my little girl, and the plays have to do with the desperate housewife education to teach girls to become mothers. Immediately after my second daughter was born, when I went to kindergarten to pick up her brother, all the girls would come to the pram to see my child, while the boys were absolutely indifferent and kept to themselves. This is wrong and is the outcome of the way we educate our children”.

According to almost all the participants, motherhood is portrayed not only as an inevitable step for women but also as a fundamental stage in improving their lives, as witnessed by Milena, who now lives in Germany with a son suffering from a suspected autism spectrum disorder, a diagnosis she has already received for herself. Milena and her wife wanted to become mothers. Milena explained, “I found myself at that one moment in my life when I was finally living a love story, sincere, honest. I had never thought of having children, and this choice did not belong to my life plans at all, but we believed that this step would have consolidated and grown our union. Now I don’t know if this choice was a response to a real desire”.

More violent, on the other hand, is the way traditional ideology operates in Italy, as Olivia, a 42-year-old Italian woman with a diagnosis of level 1 autism and two daughters (aged 6 and 4, the younger suffering from a suspected autism spectrum disorder), reminds us. She wanted to have an abortion during her first pregnancy but was prevented by the patriarchal ideology that dominates Italian obstetrical hospital wards (Crea, 2024). Indeed, in Italy, despite the fact that Law 194/1978 allows it, it is very difficult to realise this purpose: “After great difficulties, I finally found the ward. Very cold doctors gave me an appointment and gave me an ultrasound scan, during which they told me, ‘Do you see this dot?’ Why are you telling me this? [I would have liked to say but could not] ‘I didn’t come to see the dot. I’m telling you that I don’t want the dot’. However, I challenge anyone to look at the dot and remain impassive. Then I spoke to a psychologist at the hospital, who told me that, in her opinion, I wanted the children, after all. This was repeated to me by the gynaecologist, the psychologist, and the one who gave me the ultrasound. These things, at a time when I was very torn, influenced me profoundly because I am not a rock. Furthermore, I tend to be influenced, and when I feel uncertain about important choices, I can be swayed”. This testimony highlights how the figure of the idealised mother is not only a social construct but also the result of an institutional imposition that denies women the possibility of refusing motherhood and reinforces the idea that becoming a mother is both inevitable and desirable. Olivia emphasised how the idealised image of motherhood is not only upheld by social pressures but also reinforced within the family, where traditional gender roles confine women to unpaid caregiving and domestic responsibilities. The narration, in fact, continues and brings out some cursive elements in relation to regret: having lost her job, no longer having spaces of solitude in which to take refuge in herself: “For me, being a mother is a prison because I am no longer master of myself, my time, my body and the desire to do something else […] and I live with a traditional Italian male who does not even know how to put a pot on the stove”.

The problem of obstetrical violence was also emphasised by other interviewees, including Daria, who felt treated as a body and not as a person: “I didn’t threaten them with a lawsuit, but they understood that I would get there. In my birth plan, I wrote that I wanted them to ask for my consent before performing any medical procedures or treatments on me. This was not respected. Doctors are used to a certain standardised modus operandi; when a mother comes in with a little more information, she becomes a beast because she is not listened to. I understand that they are focused on the physical part and that they are interested in me staying physically healthy, but it’s not just that. You kept me physically healthy, but you destroyed me mentally […]. I didn’t have that sense of trust; it was them against me”. This testimony shows how institutional pressures contribute to reinforcing the idealised image of motherhood, generating feelings of guilt, frustration, and loss of autonomy. Even when women try to assert their choices and give consent, the system treats them primarily as “mothers”.

### 4.2. Second Thematic Area: The Guilt of Disability and Indifferent Fathers

Mother regret is also related to having children with disabilities or difficult behaviour. This was what happened not only to Olivia but also to Agnese, a 49-year-old Italian mother living in Germany, where she was working. She had a 17-year-old son who was diagnosed with autism and attention deficit hyperactivity disorder (ADHD). Managing the child had been very difficult, even more so because her husband was not supportive: “My son has always been a challenging child, always on the move, especially in the past, when he hadn’t any sense of danger. My ex-husband was not involved in this condition, so I felt all the burden of looking after my son. It has been physically and psychologically exhausting […]. Before the diagnosis, the worst thing was that the people were used to thinking that it was my fault. The message was that I wasn’t able to educate my son. Nobody thought that the problem was of the child, not me. After the diagnosis, I felt a profound sense of relief, because it was made explicit that I was not the problem, and that the child had serious problems and special needs”. Similar was the story of Loredana (47 years old), who also had two sons (ages 6 and 9, both with learning disorders and impairments in behaviour and emotion management): “It is absolutely hard to be always alone with two children with difficult characteristic [because my husband is always absent due to his work]. The little one received so many disciplinary notes this year that I wanted to dig myself a grave. Every day, the teachers would stop me outside the school. These are things I struggle with; I don’t like to feel ashamed. I live it really badly; I feel very mortified […]. It is a battle every day that I have to fight on my own”.

Daria (37-year-old married with a 2-year-old son), who quit her job to parent her child, stated, “When they say it takes a village to raise a child, it is true. It is difficult if you are alone!” The fact that the women are actually alone because of the father’s indifference has also been denounced by other participants with children without psychological problems, as narrated by Danila, a 28-year-old Italian woman living and working between Zurich and Strasbourg. She had two children (ages 1 and 3) in agreement with her husband. The first child arrived when she was still studying: “I realised I was alone. The moment I had my children, all the relationships I had changed drastically. I’m talking about friendships, but also the relationship with my husband and the relationship with my parents. You are no longer the protagonist of your own life. When you are very young, it becomes complicated. For example, for my graduation, I didn’t celebrate because I had my son […]. They say that when children are still young, regret is felt more strongly because it is more difficult, but in my opinion, I will live with regret always because who knows what it would have been like if I had not had children? I regret it a lot. I could have enjoyed the stages differently, in my opinion; I will always carry it [*weight that certainly does not concern my husband*]”.

### 4.3. Third Thematic Area: Milky Cow: You Must, You Must, You Must Because They Need It

Many participants perceived strong expectations on the part of society, resulting in negative judgements that made mothers feel strongly inadequate. Not only Agnese but also Viola, a 46-year-old married mother of two (ages 12 and 9) and a copywriter in a marketing agency, described the difficulties of managing her maternal role, exacerbated by the fact that when the little newborn cried, people expected her to know how to calm him: “‘Mummy knows’. This banal phrase is one of the worst! It’s not true; Mummy doesn’t know, just like a father doesn’t know about it! There is the expectation that you give birth, and at that moment, you are magically invested with this omniscience over your children”. A parallel form of inadequacy was described by Angela. This mother of two daughters, one of whom was suffering from epilepsy, underwent obstetrical violence and had a caesarean section: “After many difficulties, we ended up having a caesarean section. There, I felt like I was not even able to give birth, always because of the sacrificial morality that the good mother must give birth naturally […]. I then felt I was treated like a milking cow, because immediately after the birth, the nurses came in to give me the breast pump and told me, ‘You must, you must, you must, because she needs it’. And there nothing came, because between being exhausted, the operation, and everything, I started to have some milk after about a week or so. And so even there, you don’t give birth as you should, you can’t breastfeed—it’s all a house of cards that then collapses”.

Adele, a 43-year-old office worker with a 4-year-old son who was experiencing the difficulty of managing her child alone because of the death of her husband, emphasised: “I feel guilty because I realised that although I love my son very much, I also love myself very much. I come from a small town where there is a culture of motherhood based on the idea that the mother must sacrifice herself for her children and be happy to do this. But I don’t feel like that; I don’t feel happy giving myself up for my son. Then I would say to myself, ‘What a bad person you are because you love yourself as well as your child!’. It is complicated to define these feelings, also because coming from a Catholic family, the feelings of guilt are tripled”.

### 4.4. Fourth Thematic Area: You Made Your Bed; Now Lie in It

Almost all participants stated that they tried to talk about their feelings of regret with those closest to them, but they made them feel negatively judged, as Adele narrated, “I talked about my regret with my sister or my mum, and they said, ‘You did know that! Did you make your bed? Now, lie in it’. I felt terribly discomforted”. The first social token to be paid is the loss of a job, like Loredana, who had to resign her job during her first pregnancy: “I quit a full-time job when I was cornered. I would have kept that job, but the boss, who is a mother, told me, ‘Did you want children? None of your business!’” The sense of loss suffered by mothers forced to resign for their children further aggravated their regret, according to Olivia: “I miss working so much. I no longer feel like a person since I have children. If I could become a person again, regret would probably subside a little. Of course, it never goes away because a job cannot solve everything. But at least that little bit of oxygen that the job offers would make me repent for fewer hours a day, because I could think of something else”.

Interviewees also reported that they no longer felt socially recognised except in function of their being mothers. Danila, for example, has her own business, and clients often send her presents on her birthday, but since becoming a mother, the type of presents has changed: “I have stopped receiving personal presents from Italian clients, i.e., I now receive things for the children, even for my birthday. But what is the point? I love buying things for children. But even for my birthday, why? It doesn’t make much sense, you know?”

All this is related to the social expectation that mothers must have a unique identity that of being mother—so that their partners are allowed to avoid the burden of child- and household care, like Angela, who was forced to leave her job: “I definitely miss being able to manage my free time, because I never have any time, and when I have it, I have so many things I would like to do that I can hardly do half of it, and I do it badly. My partner has not given up as many things as I have, because he has always continued to work. He generally hasn’t reduced the time for his passions, and he hasn’t cancelled them as much as I have. Sometimes, I feel envy and anger. I would say, ‘But we are both parents; why do you continue to do everything you like, and I cannot?’”

Persefone, a 39-year-old widow with a daughter, perfectly summarised the strain derived by the double burden by saying, “It’s a social oppression, we women are expected to be wonder woman and perform all the time, which is not humanly possible. You can’t work as if you don’t have children; you can’t have children as if you don’t work, you can’t have the house as if you were a housewife, you can’t get everywhere”. Some interviewees stated that, despite all the fatigue involved in being a wonder woman, they see their work as a help because it allows them several positive aspects, such as not staying at home all the time, having economic independence, socialising with other people, and having money for themselves. For example, working permits Agnese to concentrate on something else: “When my child started going to school, I was happy to leave him there and then go to the office, where I could still relate with adults and have time for myself”.

### 4.5. Fifth Thematic Area: The Trap: Mum of the Year

The sense of regret ran intertwined with the perception of inadequacy, guilt, and shame, which impeded talking about it for fear of being badly judged, as Paola explained: “I find it difficult to deal with regret in the family because you can be misunderstood; it’s not easy to explain. The risk is of being judged. I couldn’t tell my husband. We love each other, but it’s something I have to carefully say”. In some cases, this is because of a sense of guilt derived from the internalised myth of the good mother, as in the case of Agnese, who stated, “In my mind, a real mother is not one who repents, that is, if one repents, she is not a real mother. So I still feel like an inadequate mother, although now we have a good relationship, I love my child and everything”. Adele also assumed the same position: “I think that maternal regret is a feeling that a mother shouldn’t have. I never imagined I would feel it, so I feel guilty. In my idea of motherhood, the perfect mother should not feel regret for having a child”.

Such a complex intertwining of sensations results in a huge sense of loneliness, as in the case of Elettra: “My prevailing feeling is loneliness, because even those who have been mothers before you, or maybe are mothers like you at that moment, it is as if they are always playing at being ‘mum of the year’. But that’s a goal you don’t aspire to, and so you don’t feel understood. The most recurring feeling is loneliness, despite having so many people around”. And loneliness is experienced as being caught in a trap, as Iris underlined: “I tell myself that I fell into the trap. It’s a bit of a trap of the patriarchy; that is, of the series go, do this, your life will surely be beautiful, full, full of wonderful things and things like that. Why, then, do all the hardships fall only on my shoulders?”

The complexity falls into ambivalence, as Persefone explained: “The love and regret of motherhood are two absolutely separate feelings, because there is nothing in the world that I love more than my daughter. It is such a visceral and deep feeling, which also comes from knowing that this living being is here by your will, and not by her choice, and that her well-being depends largely on you. To see them so vulnerable, so small, to see them forming is something incredible, which I really think cannot be explained and cannot be expressed. You know, there’s a lot of teasing from people who say, ‘You don’t have children; you can’t understand’. I tell you as a mother, it’s true; you can’t understand, you can’t understand what it feels like inside […]. And the feeling of motherhood, of love towards a child, is something that cannot be compared to anything else nothing else. And it is the love so great and the sense of responsibility so strong that make me say: ‘If I had known earlier with more conscience, I don’t think it would be an experience I would have thrown myself into’”.

## 5. Discussion

The discussion of maternal regret appears to be transversal across many cultures and nations. Heffernan and Stone (2021b) analysed responses from various countries to Donath’s research (2015), showing that in different social contexts and with different parental support policies, maternal regret is not only present but also considered taboo. Their study highlights how the role of a mother is deeply influenced by cultural narratives about motherhood prevailing in each country.

While discussing maternal ambivalence provoked a scandal in pronatalist Israel, where mothers face strong social pressure to have children (Heffernan & Stone, 2021b), the same occurred in Germany, characterised by a strong idealisation of the maternal role (Heffernan & Stone, 2021a). In Finland, however, addressing these topics appears even more taboo, as the country is perceived as a context in which it is relatively “easy” to be a mother: social policies support parents, gender equality is more advanced, and motherhood is experienced as a choice (Sihto & Mustosmäki, 2021). Nevertheless, despite these favourable conditions, cultural stereotypes persist that continue to shape maternal experiences. Similarly, in Iceland, despite being one of the most gender-equal countries in the world with innovative parental leave policies that encourage active father involvement, societal expectations and intensive mothering ideals continue to influence maternal experiences and contribute to maternal regret (Johnson & Pétursdóttir, 2024a; Gíslason & Símonardóttir, 2018).

The results of the present study highlight that maternal regret is closely mediated by cultural context and social norms, partially confirming previous research (Donath, 2015; Heffernan & Stone, 2021a). However, unlike studies conducted in other countries, the present work shows that in Italy, patriarchal pressures and the myths of the ‘good mother,’ the ‘mom of the year,’ and ‘wonder woman’ strongly influence the maternal experience, contributing to feelings of guilt, shame, anger, and isolation. Moreover, this study integrates aspects less frequently addressed in the literature, such as obstetric violence and the lack of paternal support, highlighting how these factors exacerbate maternal regret. Additional innovative elements concern the analysis of Italian participants living both in Italy and abroad, shedding light on how differences in social and cultural context shape perceptions of motherhood and associated regret. Although studies such as Johnson and Pétursdóttir (2024a) show that even in Nordic contexts with high gender equality, pronatalist pressures and intensive mothering ideals persist, the present study indicates that the specific cultural and social characteristics of Italy produce particularly complex dynamics, in which maternal regret is intertwined with historical, normative, and interpersonal factors.

Regret for having made wrong decisions belongs to the constellation of negative feelings that may accompany the experience of motherhood. According to Donath (2015), maternal regret is not only a response to everyday difficulties but also a reflection on the choices made in having children, often accompanied by a sense of loss and inadequacy, mixed with love and duty towards the parental role. This complexity is difficult to express and socially understand, so it results in the ultimate parenting taboo (Bodin, 2022; O’Reilly, 2019), which differs from other ambivalent emotions in that it reflects a deep conflict between social expectations and the desire for personal fulfilment. As the participants emphasised, neither maternal regret nor parental burnout undermines the love for one’s children. Even among women who do not experience regret about their role, ambivalence, namely, the coexistence of negative and conflicting emotions, is nonetheless regarded as a normal dimension (Takševa, 2017).

The experience of our participants was accompanied by guilt, shame, anger, and repentance or remorse, mixed with inadequacy deriving from the dominant patriarchal myth of the “good mother” consisting of the additional myths of “mother of the year” and “wonder woman”. However, despite the knowledge that these are sexist myths, they are so internalised that they constitute an important part of the suffering that accompanies maternal regret, as made explicit by Adele and Agnese. Indeed, most of the participants shared the view that having children implies dedicating oneself completely to them and their needs, especially when they are younger, and this duty is perceived as all-encompassing, so much so that it affects every aspect of life.

This type of mentality is still largely pervasive in Italy, where the percentage of working women is far below the EU average: their employment rate between 20- and 64-year-olds in Q4 2022 was 55%, while the EU average was 69.3%. Furthermore, one in five women leaves the labour market as a result of motherhood (Camera dei Deputati, 2023), and according to ISTAT calculations, they spend twice as much time on invisible work as on caring for the home and family (ISTAT, 2019). Although only 25% of our group did not work (Angela, Daria, Olivia, and Iris), similar to the results of Donath (2015), almost all participants, whether working or not, acknowledged some positive aspects of maternal love but expressed a certain type of regret. In some cases, this regret concerned the loss of freedom and the possibility of self-fulfilment in social life, while in other cases, it appeared to refer directly to motherhood itself. Many participants also felt the weight of societal expectations (Johnson & Pétursdóttir, 2024b) and perceived a fragmentation of their identity (Matley, 2020), as exemplified by Adele’s statement, “What a bad person you are”. On one hand, working mothers are exhausted by the strain that the status of a “wonder woman” imposes between work and family (“double burden”; Spagnoli et al., 2020). On the other hand, having given up work to be “mom of the year” causes regrets and exacerbates life at home, perceived as a prison. In neoliberal societies, mothers are required to reconcile simultaneously the dimensions of motherhood and working life, thereby generating inherently unattainable expectations (Rúdólfsdóttir & Auðardóttir, 2024). On the one hand, they are urged to ensure constant presence and to provide their children with the best possible education; on the other hand, they are expected to achieve high levels of performance in the professional sphere and across other domains of social life. Such responsibilities are discursively represented as expressions of individual choice, but they, in fact, emerge as the effects of a structural problem, which shifts onto the subjective level the burden of broader systemic contradictions (Boyer, 2014; Rúdólfsdóttir & Auðardóttir, 2024).

The pervasiveness of the good mother myths causes distorted communication between women generations, while the choice to become a mother is related to pandering social and family desirability, as narrated by Sara, Paola, and Milena. Almost all interviewees were unable to cope with the losses and sacrifices this role entailed, as expressed by Sara, who pointed out how women take on attitudes of falsification with regard to the accounts they gave of their maternal experience. Therefore, most participants believe that their decision could be considered unreflective, as it was only after having children that they realised what it meant (Moore & Abetz, 2018).

This constellation of thoughts and feelings is related to counterfactual thinking, which characterises all reflections on having missed opportunities for self-realisation, such as Danila, who regretted having missed important milestones in her youth. In particular, it is related to dealing with personal difficulties or those of one’s children, as with Olivia, Daria, Milena, Agnese, and Loredana, or with the loss of professional opportunities, freedom, and friendships as described by Angela (Moore & Abetz, 2018). Feeling unprepared connects with anger, which turns regret into resentment, as Sara further described, and with resentment against the mothers in the family network, as in the cases of Paola, indignant about the way Italian society raises girls to become housewives, and of Olivia, dramatically irate with the health personnel who dissuaded her from having an abortion. Having a child, therefore, came across as experiencing deceptive cultural narratives about motherhood, to which the participants reacted with anger and resentment, as stated by Elettra.

Our participant also experienced obstetric violence, which can be assumed to be a symbolic form of coercive socialisation into restrictive maternal roles, as Angela stated, should expect to be for the rest of her life. As described by Bowser and Hill (2010), the violent behaviours to which women are subjected in midwifery are dramatic: physical abuse, care provided in a dehumanising, discriminating, nonconsensual, and nonconfidential manner. Voluntary abortion happens in such a kind of environment, as described by Prandini Assis and Larrea (2022), characterised by physical violence, failure to meet standards of care, threats and criminalisation, stigma, and discrimination. Specifically, in Olivia’s case, three categories are definitely involved: the failure to meet standards of care and the following discrimination, consisting of not considering her autistic condition, which undermined her authentic needs and autonomy. Manipulative practices that invalidate the participant’s perspective are a typical phenomenon of obstetrical violence exercised by doctors, psychologists, and health workers who mystify the demands of pregnant women (Prandini Assis & Larrea, 2022), and Olivia suffered this type of prevarication. The regret in this case is also related to lowered self-esteem due to feeling easily influenced.

From the internalisation of the myths of “good mother”, “mom of the year” and “wonder woman” and the loss of personal identity, stressful emotional labour derives (Hochschild, 2012). Our results confirm what Donath (2015) indicated: maternal regret is closely linked to the management and regulation of emotions to meet social exorbitant expectations. Our participants feel compelled to conceal their emotions to conform to the patriarchal representation of motherhood, and from this psychological condition, the result is anger, which is rooted not only in the stress of balancing multiple roles but also in the inability to openly express negative emotions without feeling judged (Billotte Verhoff et al., 2023; Pedersen & Burnett, 2022). Finally, due to this substantial inauthenticity, the desire to open up to others and ask for help disappears.

But the feeling of loneliness is also related to the indifference of male partners who can go on with their lives without taking on the burden of childcare and housekeeping, as Olivia, Danila, Loredana, and Agnese denounced. On the other hand, this is due to the binary view of sexual roles that the Italian cultural tradition holds strictly. Catholic culture is firmly rooted in Italy and promotes patriarchal images of women and of men (Aloè et al., 2024; Giorgi, 2021; Indelicato & Magalhães Lopes, 2024), and the backlash is taking on increasingly dramatic profiles due to the return of right-wing governments (Shvanyukova, 2022).

All participants in the study reported perceiving pressures associated with Italian patriarchal culture. This is not only because some elements of these pressures are common across Western countries, as noted previously, but also because the participants come from the Italian cultural context and maintain family ties in Italy, and some of their partners or husbands are also Italian. In this way, Italian cultural influences and social expectations continue to play a significant role in their experiences of motherhood, even when living abroad.

Some methodological limitations should also be acknowledged, including the self-selection of participants and the lack of data triangulation, which may affect the generalizability of the results. Additionally, the study did not explore in depth certain relevant aspects that emerged during the interviews, such as experiences of obstetric violence and the temporal evolution of maternal experience, as reported by mothers with older children. Emotional ambivalence toward motherhood was also only partially addressed and deserves further consideration. A further limitation is that some participants live abroad, which introduces variability in the social and cultural context and may affect the comparability and generalisability of the findings. From a conceptual perspective, the findings are culturally nuanced, highlighting that maternal regret is not merely an individual psychological phenomenon but a socially produced and culturally mediated experience, shaped differently depending on the socio-cultural environment. This resonates with findings from Johnson and Pétursdóttir (2024a) in Iceland, showing that even in societies with high gender equality, intensive mothering discourses and pronatalist pressures remain influential. Future research could expand on these insights by including fathers who experience regret about parenthood, conducting longitudinal studies on the evolution of maternal regret, and exploring the attachment styles of parents who express such feelings. Comparative studies across different cultural and policy contexts could further illuminate how social expectations, policy frameworks, and cultural ideologies interact in shaping parental regret.

## 6. Conclusions

In this study, participants attempted to critically reflect on the Veil of Maya represented by the myth of the good mother a cultural narrative with which many Italian women have traditionally been raised and which continues to be reinforced by social representations. However, even when women recognised their feelings of regret, they still struggled with a sense of inadequacy and personal failure, which ultimately undermined their ability to integrate regret as part of an authentic maternal experience (Johnson & Pétursdóttir, 2024b; Law et al., 2021; Morris & Munt, 2019).

In Italy, feminism, as in the rest of the West, has a well-established history, but it continues to navigate challenges posed by certain strands of Catholic culture, which often promotes a binary and traditional vision of sexual roles (Giorgi, 2021; Aloè et al., 2024). This means that the myth of the “good mother”, sometimes accompanied by the parallel myths of “mother of the year” and “wonder woman”, runs parallel to the myth of the strong man who only deals with masculine things, i.e., his job and hobbies. The problem that decades of women’s history of feminist self-consciousness have failed to resolve concerns the deep unconscious internalisation of these models that permeate culture and family relations. The content of the generational mandate between mothers and daughters has not yet fully evolved and remains influenced by the ideals of the aforementioned myths. This implies that, despite decades of feminism, mothers becoming wonder women educates sons to be served and revered, without fully considering the possible effects on the future life of the woman who will build a family with him.

It is clearly necessary to re-empower women’s self-awareness groups so that they focus on this fundamental educational problem and find ways to share new forms of generational gender role mandates, moving away from the patriarchal assumptions that underlie the myth of the good mother.

Overall, the study contributes to reframing maternal regret as a complex emotional response situated at the intersection of personal vulnerability and social discourse—an experience that, rather than negating motherhood, may express a critical awareness of the tensions embedded in contemporary parenting.

## Figures and Tables

**Table 1:** Participants’ sociodemographic characteristics.

Fictitious Name	Age	Residence	Marital Status	Education	Employment	Number of Children	Age of Children	Children’s Special Needs/Disabilities
Loredana	47	Northern Italy	Married	Middle school	Yes	2	9, 6	Yes
Francesca	38	Abroad	Married	University degree	Yes	2	6, 4	Yes
Elettra	43	Central Italy	Separated (never married to father)	High school diploma	Yes	2	20, 15	No
Angela	36	Abroad	Married	High school diploma	No	2	4, 2	Yes
Persefone	39	Northern Italy	Single (partner deceased)	University degree	Yes	1	6	No
Agnese	49	Abroad	Divorced	University degree	Yes	1	17	Yes
Daria	37	Northern Italy	Married	University degree	No	1	2	No
Clara	34	Northern Italy	Married	University degree	Yes	1	2	No
Adele	43	Northern Italy	Widow	University degree	Yes	1	4	No
Milena	37	Abroad	Married (to a woman)	High school diploma	Yes	1	5	Yes
Paola	41	Northern Italy	Married	University degree	Yes	2	4, 2	No
Danila	28	Abroad	Married	PhD	Yes	2	3, 1	No
Olivia	42	Southern Italy	With partner	University degree	No	2	5, 3	Yes
Sara	35	Abroad	Married	High school diploma	Yes	1	4	No
Viola	46	Northern Italy	Married	High school diploma	Yes	2	12, 9	No
Iris	30	Northern Italy	Married	High school diploma	No	2	4, 1	No

**Table 2:** Summary of thematic analysis: areas, sub-themes, and illustrative quotes.

Thematic Area	Sub-Theme	Representative Quotes
**1. The idealised mother: pressures and hidden struggles**	Societal expectations/Idealised narratives	“You become mother because of dishonest messages. Mothers are not sincere, even with other women. I was not at all prepared for the life that was to come. Mothers closest to me—my mother, my grandmother, my mother-in-law—did not have any kind of honesty in bringing me their testimony. ‘It was all great and all natural!’ Then, when I investigated, I found out that for them, too, the story was completely different, with a suffering body, crying, haemorrhoids, hospitalisations” (Sara)
	Pressure from partners and family	“I really regretted becoming a mother when I realised that this decision was the result of the idea of motherhood inculcated by society through the family […]” (Paola)
	Conflict between personal desire and external pressures	“[…] I had never thought of having children and this choice did not belong to my life plans at all, but we believed that this step would have consolidated and grown our union […]” (Milena)
	Obstetric violence/lack of consent	“Doctors are used to a certain standardised modus operandi; when a mother comes in with a little more information, she becomes a beast because she is not listened to […]”
**2. The guilt of disability and indifferent fathers**	Child disability/difficult behaviour	“My son has always been a challenging child… My ex-husband was not involved in this condition, so I felt all the burden of looking after my son […]” (Agnese)
	Indifference of fathers/lack of support	“I realised I was alone. The moment I had my children, all the relationships I had changed drastically…” (Danila)
**3. Milky cow: You must, you must, you must**	Societal pressure/negative judgment	‘Mummy knows’. This banal phrase is one of the worst! It’s not true; Mummy doesn’t know[…]” (Viola)
	Sacrificial morality/impossibility to meet expectations	“[…]I felt like I was not even able to give birth… I felt I was treated like a milking cow, because immediately after the birth, the nurses came in to give me the breast pump and told me, ‘You must, you must, you must’[…]” (Angela)
	Double burden/role overload	“It’s a social oppression, we women are expected to be wonder woman and perform all the time, which is not humanly possible […]” (Persefone)
**4. You made your bed, now lie in it**	Loss of work/social identity	“I quit a full-time job when I was cornered. I would have kept that job, but the boss… told me, ‘Did you want children? None of your business!’” (Loredana)
	Social expectation/recognition only as mother	“I have stopped receiving personal presents from Italian clients… I now receive things for the children, even for my birthday[…]” (Danila)
**5. The trap: Mum of the year**	Internalised guilt/myth of the good mother	“In my mind, a real mother is not one who repents[…]I still feel like an inadequate mother, although now we have a good relationship[…]” (Agnese)
	Loneliness/isolation	“My prevailing feeling is loneliness, because even those who have been mothers before you[…] it is as if they are always playing at being ‘mum of the year’[…]” (Elettra)
	Ambivalence: love and regret	“The love and regret of motherhood are two absolutely separate feelings[…] To see them so vulnerable, so small… I don’t think it would be an experience I would have thrown myself into.” (Persefone)

## Data Availability

Data are available upon reasonable request.
